# Unraveling Novel Strategies: Targeting Miz1 for Degradation to Enhance Antiviral Defense against Influenza A Virus

**DOI:** 10.35534/jrbtm.2024.10009

**Published:** 2024-06-17

**Authors:** Boyu Xia, Jing Zhao

**Affiliations:** 1 Department of Physiology and Cell Biology, Dorothy M. Davis Heart and Lung Research Institute, Columbus, OH 43210, USA; 2 Department of Internal Medicine, The Ohio State University, Columbus, OH 43210, USA

**Keywords:** Miz1, Influenza A virus, Protein degradation, Antiviral host defense

## Abstract

The ubiquitin system has been shown to play an important role in regulation of immune responses during viral infection. In a recent article published in Science Signaling, Wu and colleagues revealed that transcriptional factor Miz1 plays a pro-viral role in influenza A virus (IAV) infection by suppressing type I interferons (IFNs) production through recruiting HDAC1 to ifnb1 promoter. They show that a series of E3 ligases combinatorially regulates Miz1 ubiquitination and degradation and modulates IFNs production and viral replication.

Influenza A virus (IAV) is one of the primary pathogens responsible for human respiratory infections, and a major contributor to morbidity and mortality during annual epidemics [1]. IAV infections are usually mild and self-limiting but can result in severe complications and even death, especially in individuals with underlying medical conditions or concurrent COVID-19 infection [2,3]. The efficacy of vaccinations is limited as they often target specific strains [4]. In addition to preventative influenza vaccines, treatment usually involves antiviral drugs. Apart from the traditional neuraminidase inhibitors, a novel cap-dependent endonuclease inhibitor known as baloxavir marboxil, which inhibits viral RNA synthesis, has been available for clinical use since 2018 [5]. In 2023, Via Nova Therapeutics received clearance from the FDA for the investigational new drug application of VNT-101, a small molecule inhibitor targeting influenza A nucleoprotein [6]. However, the changing nature of viruses also leads to the emergence of drug-resistant strains, thereby making antiviral agents less effective [7]. Hence, there is an urgent need for better understanding the pathogenesis of IAV infection and developing novel therapies targeting aberrant signaling pathways.

The airway and alveolar epithelium serve as the primary targets for IAV [8]. Once the virus enters and replicates within the epithelial cells, it will trigger innate immune responses, which are important for viral clearance [9]. At this time, the virus is detected by the innate immune cells expressing the pattern recognition receptor toll-like receptor 3 or 7, thereby inducing the production of type I interferons (IFNs) and IFN-stimulated genes (ISGs) that promote antiviral responses [1]. The antiviral cytokine responses, characterized by IFNs and ISGs, have been found to be absent in patients with severe IAV infections, who instead exhibited excessive production of proinflammatory factors [1]. This suggests that there may be therapeutic potential in restoring IFN signaling to rebalance the host innate immune response in severe cases of IAV infection.

A recent study by Wu et al. highlighted the involvement of Myc-interacting zinc finger protein 1 (Miz1) in regulating immune responses in the context of IAV infection [10]. The authors sought to knock out Miz1 using short hairpin RNA and the CRISPR-Cas9 system or inhibit Miz1 activation by overexpression of transcriptionally inactivated Miz1 mutant in the lung epithelial cell line (MLE-12) and lungs of mice. Their findings suggested that Miz1 suppressed the production of type I IFNs and promoted viral replication, providing new mechanistic insights on Miz1 in the suppressing antiviral immunity. Furthermore, they constructed an ifnb1 luciferase reporter in MLE-12 cells and demonstrated that Miz1 recruited histone deacetylase 1 (HDAC1) to the ifnb1 promoter, leading to histone deacetylation and eventually reducing the binding of IFN regulatory factor 3 (IRF3) and IRF7 to the ifnb1 promoter to repress its gene expression during IAV infection. Wu and colleagues also found that IAV infection reduced Miz1 protein levels without altering the mRNA level of Miz1, suggesting the posttranslational regulation of Miz1 during IAV infection. In a previous study, the authors identified Mule as the E3 ubiquitin ligase targeting Miz1 for ubiquitination and degradation in response to TNF-α challenge [11]. Here, they confirmed this finding in IAV-infected epithelial cells, and further indicated that the cullin-E3 ligase CUL4B targeted and degraded Mule, leading to the stabilization of Miz1 and suppression of IFN signaling during IAV infections (Figure 1). This novel work uncovered the crucial role of Miz1 in repressing the host immune response by inhibiting type I IFN production, and a combinatorial ubiquitin E3 ligases-directed pathway. Targeting Miz1 for degradation could be a promising antiviral strategy to fight against influenza infection. Understanding this pathway could offer a comprehensive understanding of Miz1’s involvement in the host defense against viral infections. It has been reported that Miz1 is required to maintain homeostasis of autophagy and mitophagy [12,13]. IAV was shown to induce mitophagy via nucleoprotein and matrix protein-2 [14,15]. Virus-induced mitophagy inhibits apoptosis for viral replication and enables immune escape for persistent viral infection [16]. Thus, it is likely that inhibiting Miz1 could potentially reduce viral replication by preventing virus-induced mitophagy. Other studies have implicated several high-risk factors associated with influenza mortality, such as age, pregnancy, diabetes, obesity, other underlying health conditions, and secondary bacterial infections [1,17]. Future studies should incorporate these factors into their experiment design. Emerging evidence elucidated the importance of the endothelium in orchestrating the immune response to IAV infection [9]. This study mainly focused on the role of Miz1 in epithelial cells, leaving its effect on endothelial cells unexplored. Suppression of Miz1 was also reported to be tumor-suppressive in multiple cancers such as non-small-cell lung cancer, esophageal cancer, and acute myeloid leukemia [18–20]. Despite the benefits of Miz1 inhibition, potential side effects, such as lactation defect, neural crest deficiency, and exacerbation of existing lung inflammation, were reported. Thus, caution should be taken when using Miz1 inhibition in pregnant women, children, and patients with compromised immune systems [21–23].

Posttranslational modifications are essential for intracellular signaling and protein function in respiratory diseases [24–26]. Dysregulation of the ubiquitination system is involved in the pathogenesis of influenza infection [27–30]. In this regard, the work by Wu et al. represents a huge advance in understanding the ubiquitin system during IAV infection. They rigorously identified Miz1 as a previously unrecognized target for ubiquitination and degradation, bolstering antiviral innate immunity and facilitating viral clearance. This study has established a solid foundation for future research aiming at degradation of Miz1 via ubiquitin-proteasomal pathways as well as exploration of prospective small molecules and biologics for human trials.

## Figures and Tables

**Figure 1.**
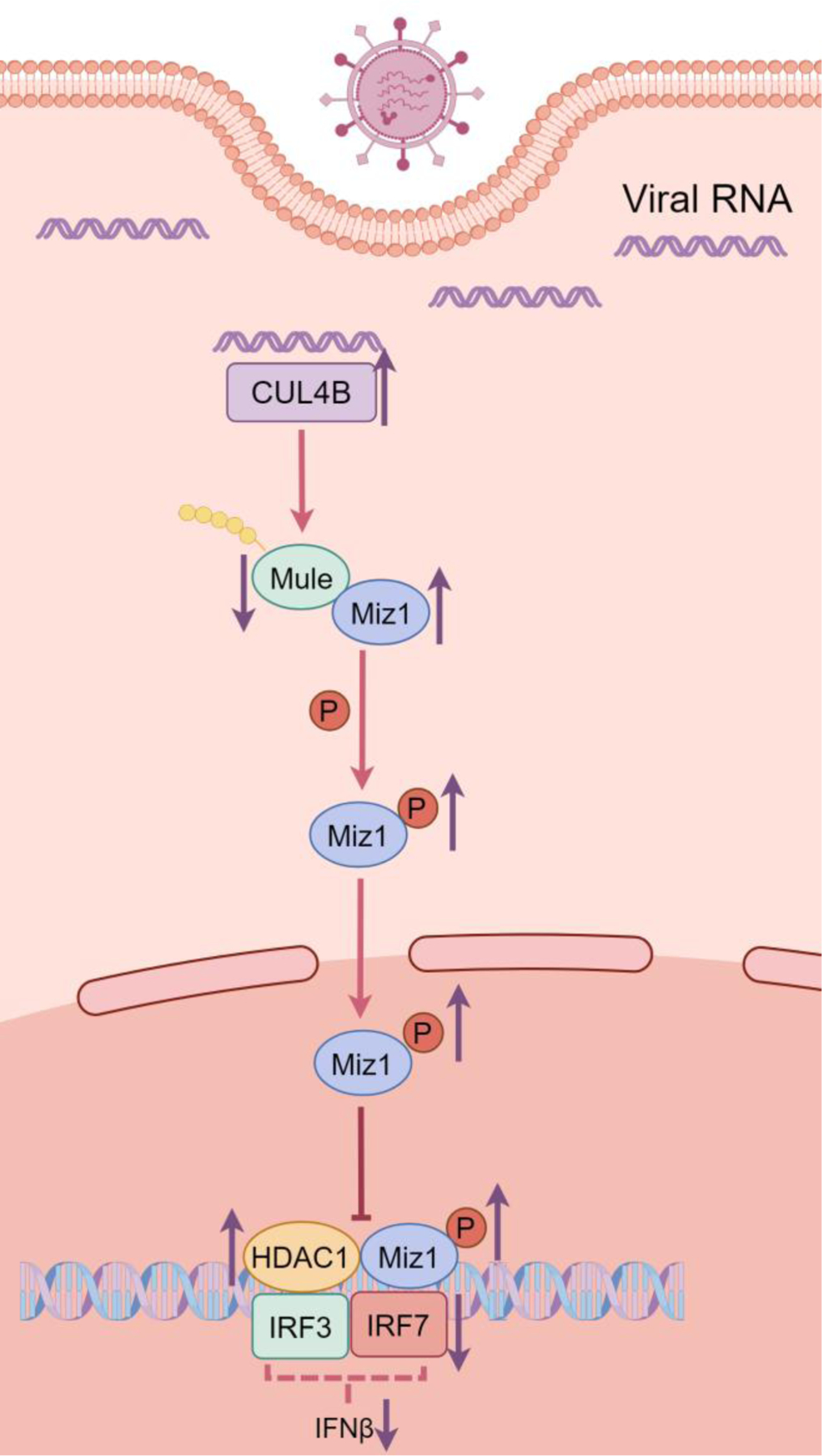
A schematic illustrating the impact of IAV-induced Miz1 expression on IFN-β transcription. IAV stimulus increases CUL4B levels, the E3 ubiquitin ligase responsible for Mule degradation. The reduction in Mule levels leads to the accumulation and phosphorylation of Miz1, inducing HDAC1 binding to the *ifnb1* promoter. This suppresses the recruitment of IRF3 and IRF7, ultimately inhibiting the gene expression and production of IFN-β. The illustration was drawn by Figdraw.
